# Towards agreement on best practice for publishing raw clinical trial data

**DOI:** 10.1186/1745-6215-10-17

**Published:** 2009-03-18

**Authors:** Iain Hrynaszkiewicz, Douglas G Altman

**Affiliations:** 1 BioMed Central Ltd, 236 Gray's Inn Road, London, WC1X 8HL, UK; 2 Centre for Statistics in Medicine, University of Oxford, Wolfson College Annexe, Linton Road, Oxford, OX2 6UD, UK

## Abstract

Many research-funding agencies now require open access to the results of research they have funded, and some also require that researchers make available the raw data generated from that research. Similarly, the journal *Trials *aims to address inadequate reporting in randomised controlled trials, and in order to fulfil this objective, the journal is working with the scientific and publishing communities to try to establish best practice for publishing raw data from clinical trials in peer-reviewed biomedical journals. Common issues encountered when considering raw data for publication include patient privacy – unless explicit consent for publication is obtained – and ownership, but agreed-upon policies for tackling these concerns do not appear to be addressed in the guidance or mandates currently established. Potential next steps for journal editors and publishers, ethics committees, research-funding agencies, and researchers are proposed, and alternatives to journal publication, such as restricted access repositories, are outlined.

## Introduction

Assessment of the reliability of published articles is seriously impeded by incomplete reporting [1]. But even if a study is impeccably reported, we usually have access only to summary information from a limited number of analyses. The availability of individual patient data, 'raw data', to the scientific community would allow many other analyses and realise a variety of benefits for science and, as a consequence, patient care. Indeed, recommendations for sharing data resulting from publicly funded research have become more common in the past few years. These include requirements of the National Institutes of Health [2], the Medical Research Council [3], and the Wellcome Trust [4]. Advocates of scientific data sharing such as the Science Commons network also strongly support this position:

'*Research data, data sets, databases, and protocols should be in the public domain. This status ensures the ability to freely distribute, copy, re-format, and integrate data from research into new research, ensuring that as new technologies are developed researchers can apply those technologies without legal barriers. Scientific traditions of citation, attribution, and acknowledgment should be cultivated in norms*' [5].

An article published in a meteorological journal describes data publication as an 'implicit part of the scientific method' [6], but very few clinical trialists currently make their raw data available. There are few strong incentives or requirements for doing so, nor is there a culture of data sharing, as has been established in other disciplines, such as the microarray [7] research community. Yet the benefits of sharing raw data have been recognised for many years. Sir Francis Galton wrote in 1901: 'I have begun to think that no one ought to publish biometric results, without lodging a well-arranged and well-bound manuscript copy of his data in some place where it should be accessible, under reasonable restrictions, to those who desire to verify his work' [8].

Sufficiently preserved and replicable data are more absolute than contemporaneously drawn conclusions and, if they are collected to address one scientific question, can later be applied for the solution of entirely different problems [9]. A key objective of this journal is to complete the scientific record by encouraging the publication of the enormous amounts of data collected over the course of randomised controlled trials [10].

The benefits of sharing clinical trial data are well documented and include reproducing and checking analyses, secondary-hypothesis testing, comparisons with previous studies, simplifying and enhancing subsequent systematic reviews and meta-analyses, and teaching [11,12]. This level of transparency also increases an article's contribution to methodological aspects of research and provides opportunities for increasing the sophistication of analyses [13]. It has further been suggested that the risk of fraud in drug trials will be reduced, as adverse events may be identified sooner [14]. And for those concerned with Impact Factors, making raw data available has also been suggested to be associated with an increased citation rate [15]. Conversely, however, the Health Canada Working Group on the Registration and Disclosure of Clinical Trial Information deemed access to raw data unnecessary, describing raw data as poorly defined and noting potential problems with consent for release of the data [16].

By providing unrestricted space, online journals such as *Trials *take the notion of sharing research data a stage further by giving authors the opportunity to publish raw data, alongside the main trial report, as supplementary material. In this editorial, we will discuss some of the challenges and opportunities for publishing raw trial data in peer-reviewed journals and propose some possible ways forward for different members of the research community.

## What guidance is available?

Because researchers have not been publishing or openly sharing raw clinical trial data in biomedical journals to any great extent, the ethical and legal issues surrounding how it should be done in an appropriate and informed way have not been thoroughly worked out. In 2001, Gunther Eysenbach, the editor of the *Journal of Medical Internet Research*, and Eun-Ryoung Sa [17] called for a code of conduct for publishing raw data, and in an article in this journal, a code was subsequently proposed for trialists and independent investigators wishing to re-analyse raw data [11]. But a widely transferable publication policy that could be adopted by many groups – authors, editors, publishers, funding agencies, ethics committees, and institutions – does not appear to have emerged.

### Journal policies

In several journals, including *Trials*, the instructions for authors include a requirement for submitting authors to be prepared to share their raw data with other scientists on request. Some journals have begun requiring access to raw data as a condition of publication, or at least transparency with regard to its availability [18,19]. The *Annals of Internal Medicine*'s 'reproducible research' initiative, announced in 2007 to increase confidence in the scientific record, set out the minimum requirements for data sharing to ensure that independent investigators could reproduce published research if they desired: 'the original protocol, the dataset used for the analysis, and the computer code used to produce the results' [19]. *Annals *asks authors to specify the extent to which they will share their data and any conditions for sharing.

Journal policies have been associated with the increased prevalence of sharing of certain types of research data, but the overall prevalence of clinical data sharing remains low [20]. And in high-throughput research and publishing environments, policing policy adherence may be beyond the resources of even the largest organisations. The growth of online open-access publishing, often without restrictions on supplementary material, should, in principle, provide the platform for publishing raw data. But several issues commonly arise as barriers to publishing raw data in journals.

### Confidentiality and anonymity

A key concern for any use of personal health information must be anonymity [21]. Publishing data that have arisen from the doctor-patient relationship whilst preserving the privacy of individuals – unless explicit consent has been obtained – remains a challenge, particularly in light of statutes such as the Health Insurance Portability and Accountability Act (HIPAA) in the US and the Data Protection Act in the UK. For example, the confidentiality policy of the *BMJ*, a UK-based publication, has been established to comply with the Data Protection Act [22]. However, in an increasingly global publishing industry, universally agreed-upon definitions as to what constitutes anonymised (or 'de-identified') information arising from personal health records do not appear to have been established.

Publishing and editorial support groups' privacy policies are reasonably clear with regard to individual patient case histories and small case series [23,24], but for larger clinical studies, in which the raw data can still contain detailed information about individuals, there seems to be a lack of appropriate guidance on how a researcher would tackle this issue. If a data set can be anonymised so that neither the patient nor anyone else could identify an individual, then data are no longer 'personal', negating the issue of consent for publication. The benefits of access to the full data set for the scientific community could arguably outweigh the small risk that an individual within a data set might be identified. Complete anonymity can be difficult to achieve with certainty, as noted by the International Committee of Medical Journal Editors [24]. But for the majority of clinical trial data sets, confidentiality would rarely be a concern if the data consisted of very common baseline demographic information and one additional variable (such as pain scores in a trial of treatment for pain). The HIPAA provides an explicit list of 18 items that need to be removed from patient information in order for it to be considered anonymous for sharing (rather than publishing) information among the Act's 'covered entities' [25].

### Ownership

Ownership of raw data sets is also a contentious issue. Although trialists may in some cases be the legal owners of their data sets, whether it is morally right for researchers to keep potentially useful information arising from personal health records is debatable [11]. Moreover, where data have been generated via a collaborative effort by multiple researchers (often during the course of employment), potential infringements of third-party rights may also need to be considered. However, as noted in the Research Information Network's study, published in June 2008, researchers themselves are often unsure of who owns their data [26]. With regard to publishing data, the Association of Learned and Professional Society Publishers issued a statement in 2006, supporting sharing of raw data sets among scholars as a general principle [27]. It recommends that publishers separate supporting data from the article itself and not require transfer of copyright as a condition of publication.

## Other challenges and opportunities

Medical journals must often subscribe to more stringent publication policies for peer-reviewed articles compared with other means of disseminating information, such as the lay press, so it is reasonable to assume that the same should apply for raw data sets. Publishing raw data from 'historic' trials is particularly problematic as it is unlikely that allowances for such reuse of the data will have been made in patient consent forms for trials conducted many years ago. And this may still be the case for newly approved trials. Where explicit consent for publication of data is not obtainable, approval for release of data is desirable, but from which person or organisation is generally unclear.

This is highlighted by the limited access to data sets of the US National Heart Lung and Blood Institute (NHLBI) when there is an absence of clear approval by study patients for data sharing in older studies. The NHLBI institutional review board requires that there be agreement by data recipients that they will not try to identify any individuals and that an institution will vouch for the integrity of the process. If consent to release data is obtained from patients, this extra step is not needed [28,29]. The practice of obtaining consent retrospectively presents its own problems. The value of a data set is diminished if one or more patients decline consent or cannot be traced, and the requirement to obtain retrospective consent also lacks a consensus. However, some ethical guidelines on the management of clinical trial data can require informed consent from the patient for any reuse, redistribution, or publication of the data from the trial [30].

## Possible ways forward

In light of all of these considerations, it seems a policy that could be followed by many groups, including editors, the industry, ethics committees, and research-funding agencies, would be an important step forward. It has been recommended that academic institutions take the lead on data-sharing initiatives by providing incentives, funds, and publication policies [31], but we would argue that there are roles for a number of parties who are affected by data sharing and publication.

In September 2008, the editors of this journal convened a meeting with relevant members of the scientific and publishing communities to discuss these issues. Whether the data being published are from previously published trials or from proposed or ongoing research emerged as a key consideration. Publication of data from historic trials is likely to need a case-by-case assessment taking into account any special circumstances surrounding a trial, and who is best placed to give this advice or approval remains open to debate. Nevertheless, some suggestions for other members of the community likely to be involved in prospective data publication are outlined below.

***Ethics committees:*** Encourage researchers to include plans to publish data in trial information sheets and discuss the safeguards in place to protect patient privacy.

***Research-funding agencies:*** Give greater scrutiny to data-sharing plans and monitor their enforcement.

***Journal editors and publishers:*** Recommend that authors prepare data in line with an agreed-upon standard (what this is requires further consideration). Encourage deposition of data in the journal or suitable third-party repository as part of the submission process, potentially via an accession number system, as is established for trial registration.

***Trialists:*** Obtain explicit consent for publication of suitably anonymised raw data as part of patient recruitment procedures.

### Sharing data without publication

Different publication standards might be required for individual studies (for example, those involving sensitive or rare conditions) or studies involving certain populations. There are circumstances in which all the raw data cannot be in the public domain and these different standards could incorporate differing levels of access to certain types of data and/or embargoes on access. And where publication of trial data in journals is not possible, the involvement of a suitable third party could be considered, with appropriate restrictions on access where necessary. Accreditation schemes for researchers wishing to access data, as instigated at the NHLBI, would be a logical part of this process.

Online repositories for data sets, such as the Dataverse Network Project [32], do exist, and the number of institutional repositories has increased markedly since 2002 [33]. In the social sciences in particular, a number of data archives have been established [34]. But regardless of what restrictions on access are in place, a researcher wishing, for example, to find all currently available data sets from trials of a particular drug in a particular condition would face significant challenges given the poor support for sharing clinical data and lack of consensus on the appropriate repository.

## Concluding remarks

Can we agree on best practice for publishing raw clinical trial data? An important step forward will be to prepare widely agreeable guidance on preparing raw data for publication and recommendations for researchers on handling retrospective and prospective trial data: a challenge the editors of this journal are currently undertaking. However, mandates will need to be established at the funder, institution, or journal level to facilitate and influence cultural changes. The US Food and Drug Administration (FDA) Amendments Act of 2007, for example, now requires disclosure of trial *results *supporting FDA-approved drugs within a year of drug approval [35]. Once there is increased prevalence of data sharing and publication in the clinical trials community, the further challenges (not specifically addressed in this article) of how data sharing is to be standardised and what in fact constitutes 'the raw data set' can subsequently be tackled.

## Postscript

Any members of the clinical trials community interested in participating in this journal's initiative to agree on best practice for publishing raw clinical data are encouraged to contact the editorial office at editorial@trialsjournal.com.

## Abbreviations

FDA: Food and Drug Administration; HIPAA: Health Insurance Portability and Accountability Act; NHLBI: National Heart Lung and Blood Institute.

## Competing interests

DGA is editor-in-chief of *Trials*. IH is employed by BioMed Central Ltd, which publishes *Trials*, and receives a fixed salary.
